# Problems and solutions of personal protective equipment doffing in COVID-19

**DOI:** 10.1515/med-2020-0172

**Published:** 2020-07-02

**Authors:** Lin Cheng, Lei Chen, Li Xiao, Jianping Zhang, Yilian Cheng, Lian Zhou, Yu Peng, Lei Liu

**Affiliations:** Department of Radiology, Southwest Hospital, Army Medical University, Chongqing, 400038, China; Department of Geriatric, Southwest Hospital, Army Medical University, Chongqing, 400038, China; Department of Nephrology, Southwest Hospital, Army Medical University, Chongqing, 400038, China; Department of Cardiology, Southwest Hospital, Army Medical University, Chongqing, 400038, China; Department of Nursing, Southwest Hospital, Army Medical University, Chongqing, 400038, China

**Keywords:** coronavirus disease 2019 (COVID-19), origin of the pandemic, occupational exposure

## Abstract

The progress of the coronavirus disease 2019 (COVID-19) pandemic is still severe. While everyone has been striving very hard to combat the pandemic, some healthcare professionals have shown varying degrees of infection symptoms and even died. The Chinese Army Medical Aid Team arrived in Wuhan on January 25, 2020, and quickly entered the battle against the pandemic after a short and rigorous training. As one of the earliest medical teams to enter the pandemic area, researchers have been exploring effective infection control measures that are currently in practice. Through observation and research, it has been noticed that layers of protective equipment have a hidden danger of infection while protecting the safety of the personnel. The members of each medical team have typically focused on the standard use of personal protective equipment (PPE). However, after a long period of intensive diagnosis and treatment in clinics and due to physical exertion and tiredness, problems such as improper operation are prone to occur during the tedious PPE doffing, thereby producing a relatively high risk of infection. This study primarily analyzes PPE doffing procedures, existing problems, and measures for improvement to explore methods to improve PPE donning and doffing and reduce the risk of infection.

## 1 Introduction

In December 2019, the outbreak of coronavirus disease 2019 (COVID-19) emerged. This virus mainly transmits through respiratory droplets and contact leading to the rapid transmission and the extensive number of infected people [1]. The typical clinical symptoms of COVID-19 are fever and dry cough, while a small number of cases are with nasal congestion, tears, pharyngeal pain, diarrhea, and other symptoms [1]. Severe challenges exist in pandemic prevention and control. Although the level of hospital infection control in China has been greatly improved in recent years, a series of guidelines for prevention and control based on the characteristics of COVID-19 have been issued during the pandemic. However, as of April 8, 2020, 32 healthcare professionals in China have died after they were infected due to occupational exposure, from then on, no healthcare professionals died from infection were reported. Therefore, to prevent the pandemic from spreading further and to ensure the safety of frontline medical personnel, standardizing the procedures for personal protective equipment (PPE) donning is one of a series of important measures for prevention and control [2]. Disposable coverall suits, face shields, and other PPE for medical purposes effectively protect medical personnel from infection when in contact with blood and bodily fluids of patients with infectious diseases [3]. However, after completing intensive work, it is very important to safely remove the protective clothing and other PPE according to the appropriate procedures. Any person who is slightly inattentive when doffing PPE by not following the standard implementation or operation can increase the occupational exposure and the risk of infection. The Chinese Army Medical Aid Team performed strict training and control regarding PPE donning and doffing during the COVID-19 pandemic and noticed problems. We performed an in-depth analysis of the doffing procedures followed by the proposal of improved measures to address the corresponding problems. This study aims to reduce the risk of healthcare professionals being infected with COVID-19.

## 2 Procedure for PPE donning and doffing

**Figure 1:**
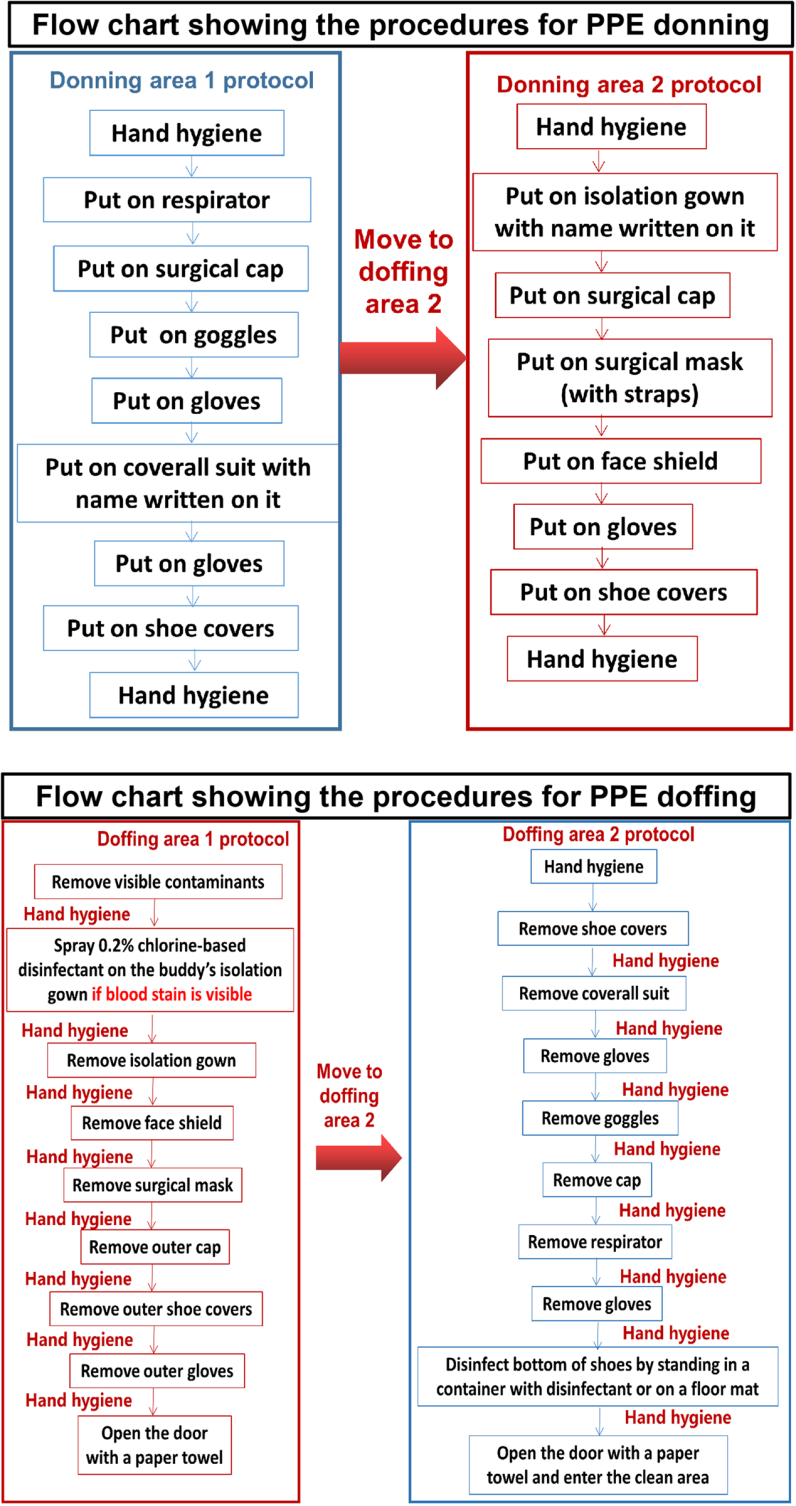
Flow chart showing the procedures for PPE donning and doffing.

The Chinese Army Aid Medical Team gained extensive experience in combating the emergence of severe acute respiratory syndrome (SARS) and swine flu (H1N1) pandemics. As a result, they redesigned the procedures for donning and doffing of PPE according to the characteristics of the novel coronavirus SARS-CoV-2 (Figure 1). A model of two-person assistance (the buddy system) and mutual supervision was adopted. In addition, the SkyEye monitoring system was installed in nurse stations, physician offices, PPE donning areas, and PPE doffing areas to observe and monitor in real-time using arranged shifts of infection-control teams on a 24 h basis. Hence, staff are reminded in a timely manner of the precautions for donning and doffing PPE to ensure their safety.

## 3 Problems encountered in PPE doffing

When the infection-control team reviewed and analyzed monitoring videos of PPE donning and doffing, it was found that although the steps of donning were complicated, everyone managed to focus on the donning standards layer by layer without major problems, and the donning areas were maintained as clean environments. The PPE were also clean and sterile, and therefore, the risk of COVID-19 infection was low. However, during PPE doffing, due to the long hours of intensive diagnostic and treatment duties and the extremely extensive physical exertion, staff were susceptible to mistakes due to fatigue, causing themselves to become more prone to infection. After prolonged contact with COVID-19 patients, the PPE were exposed to high-risk environments and became seriously contaminated materials [4]. Incorrect doffing of PPE can be more fatal than direct occupational exposure to the virus, because medical staff may be infected while doffing PPE without realizing it, whereas direct exposure is always dealt with immediately. The following subsections summarizes the analysis of the problems in the PPE doffing procedures.

### 3.1 Inadequate implementation of hand hygiene

The seven-step handwashing process was not strictly followed, and there were missed steps and insufficient time for hand-rubbing (<15 s). Due to the long hours and overloads in duties, staff often felt physically and mentally tired. The total number of required handwashing steps for completing the procedure in the two doffing areas was 16 times, which possibly reduced the strict compliance of staff in performing adequate handwashing.

### 3.2 Too close a distance for the disinfectant spraying

**Figure 2:**
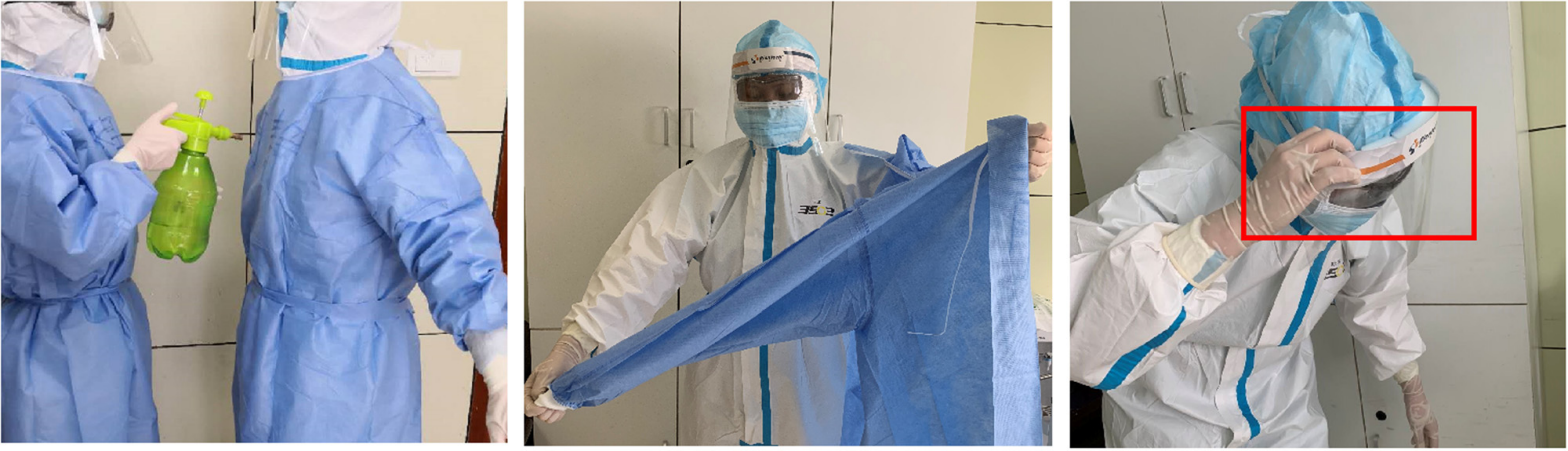
A representative image showing incorrect methods for disinfectant spraying, the isolation gown moving, and face shield removal.

The two people doffing PPE together (the buddies) are required to spray a 0.2% chlorine-based disinfectant on each other. As shown in Figure 2, when the disinfectant was sprayed too close to the subject, the atomization effect was not ideal, and the person did not avoid the head and neck region.

### 3.3 Too vigorous movements when removing the isolation gown

As shown in the middle picture of Figure 2, the movement was too vigorous when removing the blue isolation gown. Hence, the gown was not gently rolled away from the body to contain the soiled outside surface inwards.

### 3.4 Improper removal of the face shield

As shown in the last picture of Figure 2, touching the side edge or front surface of the face shield when removing it caused contamination.

### 3.5 Contamination during surgical mask removal

**Figure 3:**
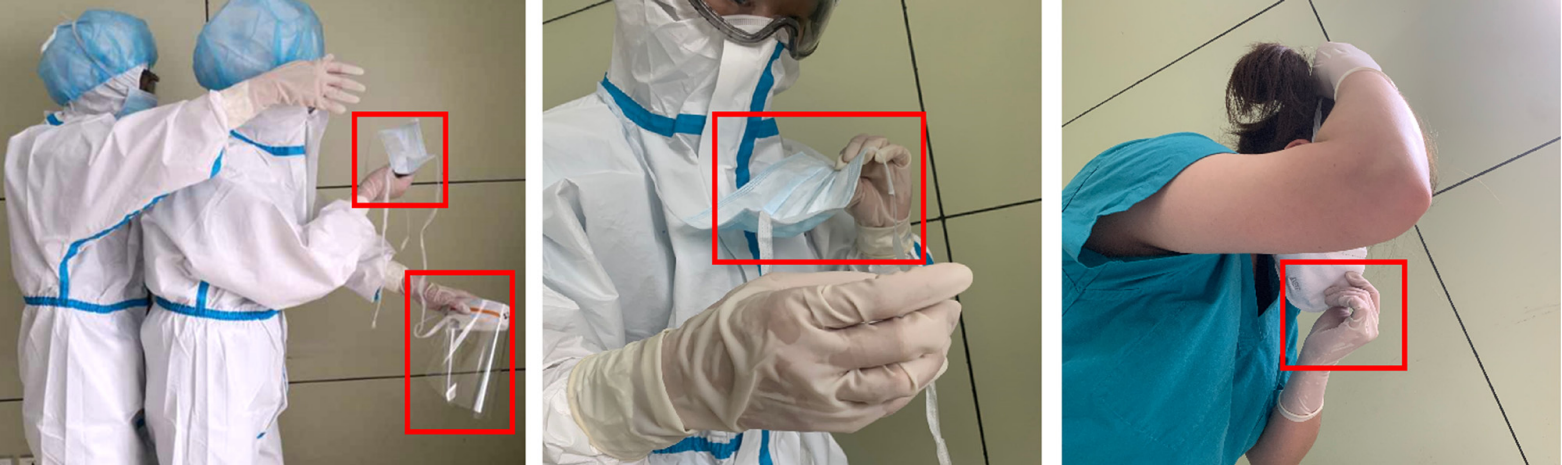
A representative image showing that incorrect methods have a high risk to contaminate surgical mask, the disposable coverall suit, and face shield.

As shown in Figure 3, when the staff member untied the upper tie straps of the surgical mask, it fell down and contaminated the chest area of the disposable coverall. Therefore, the effect of wearing the isolation gown and lowering contamination of the doffing area was lost. Other mistakes included touching the front surface of the surgical mask or the respirator (the last two pictures of Figure 3).

### 3.6 Inappropriate doffing of surgical caps

Several instances were noted when the front surface of the surgical caps was touched during doffing.

### 3.7 Too vigorous movements when removing the disposable coverall suit

**Figure 4:**
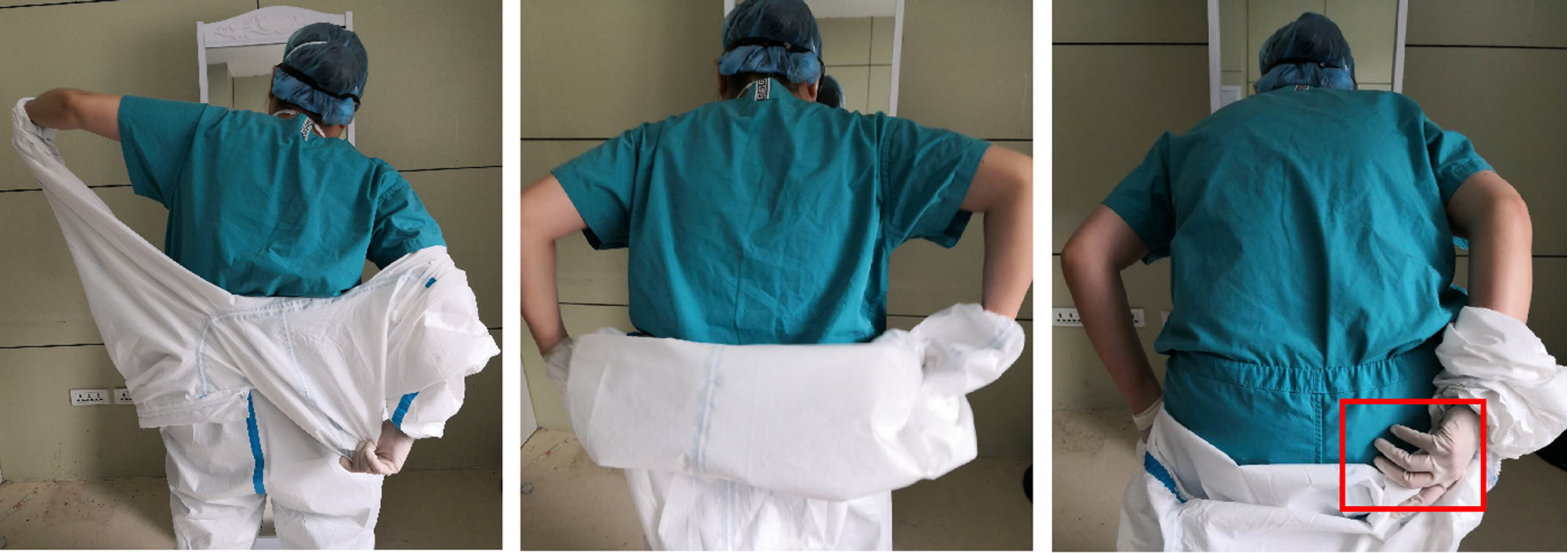
Representative images showing the vigorous movements during the removal of the disposable coverall suit, with the person’s gloved hand touching and contaminating the inner medical scrub.

As shown in Figure 4, the staff member removed his disposable coverall suit using vigorous movements and obvious pulling. Therefore, the coverall suit was not tightly rolled down to contain the outside of the suit inwards. He also touched his inner medical scrub with his gloved hand.

### 3.8 Inappropriate removal of gloves

**Figure 5:**
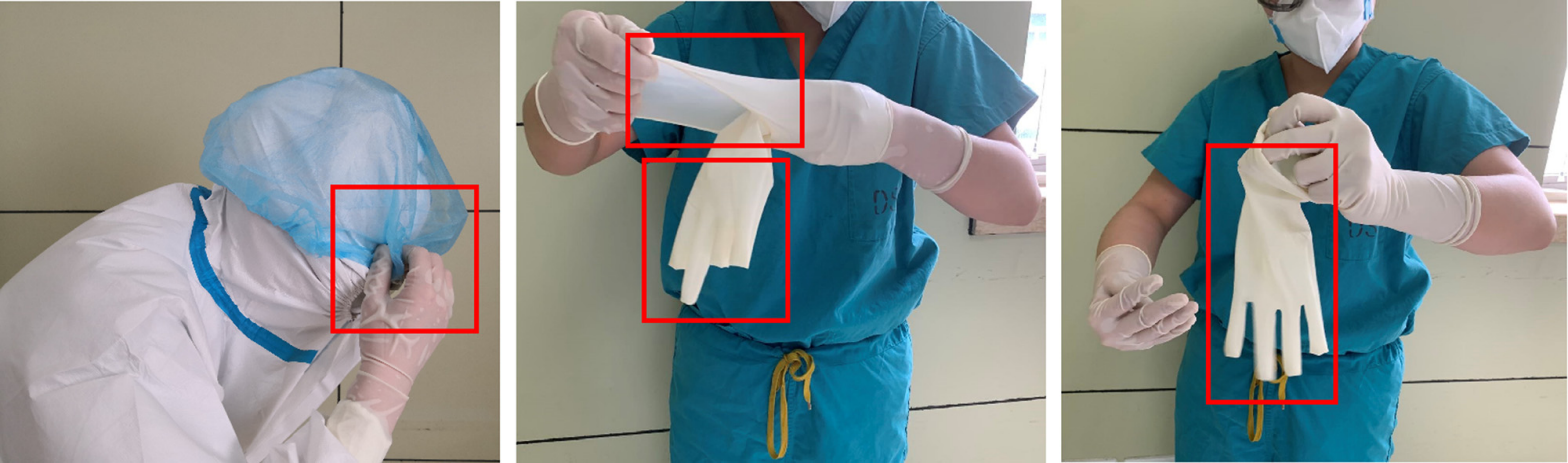
Representative images showing an inappropriate removal of surgical gloves.

As shown in Figure 5, the staff member removed his gloves in a vigorous movement and did not peel the gloves away from both hands inside out or wrap them together.

### 3.9 Other issues

Other mistakes were observed in the SkyEye videos. For example, two staff members were found to be chatting loudly and staying for an extended period in the doffing area, which would increase the possibility of cross-infection. Also, some staff members only wrote their names on the isolation gown, but not on the disposable coverall suit. In addition, it was found that medical staff pressed garbage bags vigorously when collecting medical waste to exhaust the air, which may produce aerosols that will contaminate the environment.

## 4 Summary of the important points for PPE doffing

### 4.1 Standardizing procedures for hand hygiene

Staff should strictly implement the steps and the time needed for the seven-step handwashing technique. The infection-control expert group requires that all staff must rub their hands for more than 15 s.

### 4.2 The donning and doffing of PPE with another staff member

Each person should don and doff their PPE with a buddy to help each other. A proper distance should be maintained when spraying the chlorine-based disinfectant to allow for full atomization and to achieve effective sterilization. Moreover, the spraying of disinfectant should avoid the head and face to prevent the disinfectant from irritating the respiratory tract and mucous membranes of the person.

### 4.3 Removal of the surgical mask

The removal of a surgical mask should be performed by the buddy. Both persons should face the same side, with one standing behind the other. The person in the front should slightly lower the head while the person at the back should untie the lower tie straps of the surgical mask before untying the upper tie straps of the surgical mask of the person in the front. This should be followed by handing over the straps to the person in the front and letting him/her remove and discard the surgical mask.

### 4.4 Procedures for doffing the disposable coverall suit

In strict accordance with the standard procedures for doffing the disposable coverall suit, the staff member should slowly and gently remove the PPE. Implementation of immediate hand hygiene is necessary after accidental contacts with a contaminated surface, such as the side edge or front surface of the face shield and the front portion of the surgical mask and hat before implementing subsequent operations. The doffed PPE should be disposed of gently into a medical waste container.

### 4.5 Proper scrub wearing

The scrub top should be tucked into the scrub pants properly to prevent contamination.

### 4.6 Remain vigilant

All staff should remain vigilant at all times when doffing PPE and should not overlook the procedures because they think they have mastered the doffing procedure. Unnecessary conversation should be avoided in the doffing area. All staff should leave the doffing area immediately after the removal of PPE.

### 4.7 Disposal of medical waste

Doubled biohazard trash liners should be used to collect medical waste in the medical waste containers. Medical waste should be sealed before collecting the garbage bag to avoid excessive handling and increasing the risk of contamination.

## 5 Improvement measures

### 5.1 Emphasize training

The tasks for combating the COVID-19 pandemic are intense and critical. Medical teams in China have engaged in combat as quickly as possible, and their training in PPE donning and doffing was brief and rushed. Online training modes should be adopted because physically gathering in a training area is not appropriate during this pandemic [5]. The WeChat social media app can be used. Relevant theoretical knowledge, the latest expert consensus, and standard procedures can be shared in WeChat groups. Existing problems should be analyzed and discussed to consolidate the related theoretical knowledge through online questioning. Video-based online teaching can be also used for steps that receive multiple questions. On-site special training can be applied for people who experience difficulty in mastering the steps. Solid knowledge and skills regarding personal protection are the most basic and effective ways of self-protection during this pandemic.

### 5.2 Focus on supervision and self-inspection

The guidance and supervision of the infection-control team are indispensable throughout the entirety of the prevention and control measures to ensure that healthcare professional protective measures and the procedures of diagnosis and treatment meet management requirements. By using the SkyEye monitoring system, the infection-control team can monitor the procedures of PPE donning and doffing in real-time to remind healthcare professionals at any time to avoid contamination. Alternately, the monitoring video can be played back to staff so they can identify their own mistakes and correct themselves accordingly.

### 5.3 Emergency response plan for exposure during the doffing of PPE

The emergency response plan for exposure to contaminated PPE includes the following steps: (1) immediately suspend the doffing procedures once exposure occurs. The exposed area should be immediately disinfected by the buddy in the doffing area. (2) If exposure occurs to the face or other skin surfaces, immediately apply 75% alcohol or ethanol-containing quick-drying hand sanitizer to wipe the exposed skin on the face or other area for 2 min. If exposure occurs to ocular mucosa, repeatedly rinse with normal saline and apply anti-infective eye drops. If exposure occurs to the oral mucosa, gargle with 75% alcohol once for 2 min, followed by gargling with normal saline three times. (3) Continue doffing other PPE according to the procedures. (4) Shower and change clothes. (5) Finally, report the relevant information to the infection control team.

## 6 Summary

The multiple transmission routes of SARS-CoV-2 cause healthcare professionals to encounter an enormous risk of infection during diagnostic and treatment procedures [4]. Due to this risk, strengthening the supervision of PPE doffing is required. This supervision work was primarily completed by an infection control and management team composed of professional infection-control experts. They provided real-time supervision and guidance regarding protection for healthcare professionals using a monitoring system and on-site tracking. Due to the emergency situation of the pandemic, the monitoring system did not cover all hospitals. However, the establishment of infection management teams to perform real-time management of the daily protection measures of healthcare professionals is vital in protecting the safety of physicians and patients and avoiding cross-infection.

The medical team involved in this evaluation primarily treats patients with critical cases of COVID-19; therefore, they were required to meet the biosafety level 3 requirement for PPE. Based on the protection experience gained in the SARS and H1N1 pandemics, slight over-protection can effectively protect the safety of healthcare professionals during a pandemic, and this fact is well accepted by healthcare professionals. Therefore, under the guidance of infection control experts, a higher level of protection above the World Health Organization (WHO)’s recommendation was implemented [6]. During the process of observation and research, it was found that medical team members should focus on standardized PPE donning. However, it should be noted that the selection of PPE to adequately fit the body shape of the person is necessary to ensure comprehensive coverage without leaving gaps, and the staff should remain dressed this way when treating patients [7]. In addition, the doffing of PPE after the shift specifically needs more attention. This study adopted the buddy system throughout the procedures of donning and doffing PPE, which showed advantages in PPE doffing. Removal of the isolation gown and surgical mask with the assistance by the buddy avoided contamination and exposure to the greatest extent. The buddy system in donning and doffing can prompt each person to remind each other of possible problems. The buddy can also assist with disinfection treatment in the event of exposure, achieving the purpose of mutual supervision and cooperation. To avoid exhaustion of healthcare professionals, the working hours when wearing PPE should generally not exceed 4 h. Nevertheless, after a shift wearing heavy and sealed PPE, healthcare professionals often sweat a lot and are exhausted, and they might experience syncope. Under this circumstance, the buddy system can provide mutual assistance.

In summary, although it is important to pay attention to donning PPE, nonstandard procedures in PPE doffing are more likely to cause exposure to the infectious virus. Therefore, the improvement and strict control of PPE doffing by using reasonable procedures and existing resources are effective means to ensure the safety of healthcare professionals and to reduce the risk of cross-infection.

## 7 Limitations

In this study, a control study did not be conducted to quantify how proper PPE techniques would lower the infection risk in healthcare staff because of ethics. We hope that this issue could be addressed using other methods in future studies.
